# Colorectal Cancer Patients with Low Abundance of KRAS Mutation May Benefit from EGFR Antibody Therapy

**DOI:** 10.1371/journal.pone.0068022

**Published:** 2013-07-09

**Authors:** Shaorong Yu, Xia Xiao, Jianwei Lu, Xiaoping Qian, Baorui Liu, Jifeng Feng

**Affiliations:** 1 Research Center for Clinical Oncology, Nanjing Medical University Affiliated Cancer Hospital, Jiangsu Cancer Hospital & Jiangsu Institute of Cancer Research, Nanjing, Jiangsu Province, China; 2 The Comprehensive Cancer Center of Medical School of Nanjing University Affiliated Drum Tower Hospital, Jiangsu Key Laboratory for Molecular Medicine & Clinical Cancer Institute of Nanjing University, Nanjing, Jiangsu Province, China; Faculty of Medicine, University of Porto, Portugal

## Abstract

Epidermal growth factor receptor monoclonal antibody was approved for treatment of metastatic colorectal cancer patients carrying KRAS wild type DNA. However, recent studies showed that patients with KRAS G13D mutation may benefit from EGFR antibody therapy. In this study we tried to explore whether the abundance of KRAS mutation could affect the efficacy of EGFR antibody therapy. We firstly established a PNA-PCR method which could calculate the percentage of KRAS mutation in total DNA and proved its ability on 47 colorectal cancer samples bearing KRAS mutations. Then we analyzed the correlation between the abundance of KRAS mutations and efficacy of EGFR antibody therapy in another 35 metastatic colorectal cancer patients. We proved that PNA-PCR assay could calculate the abundance of KRAS mutation and the percentage of mutant DNA in tumor cells varied a lot (10.8%∼98.3%) on the 47 colorectal cancer patients. The efficacy of EGFR antibody correlated with the abundance of KRAS mutations: in the KRAS mutation less than 30% group, the disease control rate was 44.4% (4/9); the disease control rate of 30∼80% group was 5.6% (1/18) and the >80% group was 12.5% (1/8) (P = 0.038). In summary, our study showed that PNA-PCR method could easily detect the percentage of KRAS mutation in tumor cells and colorectal cancer patients with low abundance of KRAS mutation might benefit from EGFR antibody therapy.

## Introduction

Epidermal growth factor receptor (EGFR) monoclonal antibody was approved for treatment of metastatic colorectal cancer patients without KRAS mutations. KRAS mutations are described to occur in approximately 40% of colorectal cancer and most of them (90%) occur in codon 12 and 13 [1], [2]. Several phase II and III clinical trials have proved a lack of response to anti-EGFR therapy in the presence of KRAS mutations [3], [4], [5]. These results led the European Medicines Agency and the Food and Drug Administration to limit anti-EGFR treatment only to patients carrying wild-type KRAS tumors in 2009 [6].

However, recent studies showed that KRAS G13D mutation and codon 12 mutations are actually not created equal in predicting clinical outcomes of anti-EGFR therapy in metastatic colorectal cancer (mCRC) patients [7], [8]. Patients carrying KRAS G13D mutation could still benefit from cetuximab treatment [9]. In a multivariate analysis, patients with G13D mutation tumors treated with cetuximab had longer overall survival (median, 7.6 months vs 5.7 months) and longer progression-free survival (median, 4.0 months vs 1.9 months) than other KRAS mutant tumors [10]. Several meta-analysis also got similar results [7], [11]. Other than that, a pooled analysis of three trials showed that specific mutation in KRAS codon 12 also had different impact on treatment efficacy in colorectal cancer patients and tumor bearing a KRAS G12D mutation showed a strong trend to a more favorable outcome comparing to other codon 12 mutations [12]. All of these studies showed us that not all KRAS mutant tumors were resistant to anti-EGFR therapy. As researches continued, it was not sufficient to roughly differentiate patients with KRAS mutation status.

Since tumor had great heterogeneity, different tumor tissues may also have variable abundance of KRAS mutant tumor cells. Since every screening method had its detection sensitivity, when the tumor KRAS mutation’s proportion reduced to a certain extent, this tumor sample might be recognized as KRAS wild type. As more and more highly sensitive screening methods are established, more tumor specimens will be classified as KRAS mutant ones [13], [14]. We still don’t know whether these samples will be resistant to EGFR-antibody therapy. In this study, we hypothesized the abundance of KRAS mutation might also affect the efficiency of anti-EGFR treatment. Actually, similar with our hypothesis, in 2011, Zhou et al found that high abundance of EGFR mutations had higher objective response than low abundance in non-small-cell lung cancer treated with gefitinib [15].

In our former study, we established a PNA-PCR (peptide nucleic acids-PCR) method which could suppress the amplification of KRAS wild type DNA. In this study, we first modified this PNA-PCR method to make sure that it could totally suppress amplification of KRAS wild-type DNA and confirmed its suppression ability on 47 tumor samples bearing KRAS mutations. Then we quantified and calculated the proportion of KRAS mutant DNA in tumor tissues and found that the proportion varied a lot. Finally, we compared the relationship between KRAS mutation abundance and the efficiency of cetuximab in another 35 metastatic colorectal cancer patients.

PNA-PCR method was established before in our laboratory [16], and in this study we made minor adjustments in order to make sure it could quantify and calculate the proportion of KRAS mutant DNA. This method could amplify KRAS mutant DNA exclusively and could quantify KRAS mutant DNA simultaneously. In the same time, we could also quantify the amount of total DNA (both KRAS mutant and KRAS wild-type DNA) by regular quantitative PCR (PCR without PNA). Then we could easily calculate the percentage of KRAS mutant DNA in total DNA.

## Materials and Methods

### Cell Lines and Patients

Colon carcinoma cell lines SW480 and SW116 (purchased from BIOK&KM, China) were used in this study. Cell line SW480 contains a homozygous KRAS codon 12 mutation (GGT→GTT) and SW116 harbors wild-type KRAS gene. A total of 47 colorectal cancer patients’ FFPE specimens whose KRAS status was proved to be mutant on either KRAS codon 12 or codon 13 were obtained from the Drum Tower Hospital from November 2008 to December 2010. Another 35 colorectal cancer patients with KRAS mutation were collected from Jiangsu Cancer Hospital from 2006 to 2010. Different from patients above, all of these 35 patients received cetuximab treatment. Informed written consent was obtained from all patients before treatment started. All treatment decisions were made by the treating physicians prior to design of the study. The study was approved by the institutional ethics committee of hospitals (Ethics Committee of Drum Tower Hospital and Ethics Committee of Jiangsu Cancer Hospital) before this study was conducted.

### DNA Extraction from SW480, SW116 and FFPE Tissues

After manual macrodissection of FFPE tissues, the hematoxylin and eosin-stained sections of FFPE tissue were reviewed by three experienced pathologists to evaluate the abundance of tumor cells. The detailed evaluation method has been described before [17]. The sample’s tumor cell percentage is the mean of the values obtained by the three pathologists. Genomic DNA was isolated from FFPE samples, SW116 and SW480 with Recover All total nucleic acid isolation kit (catalog no. AM1975, Ambion) according to the manufacturer’s instructions. A negative control was performed to examine the possibility of contamination. DNA concentrations were determined by UV (260 nm) spectrophotometer.

### PNA-PCR Plus Pyrosequencing

PNA-PCR and pyrosequencing conditions have been described previously except for PCR master mix [16]. Briefly, the PCR master mix contained final concentrations of reagents as follows:1×SYBR Premix Ex Taq Mix (TaKaRa, code DRR041A), 0.15 umol/L forward and reverse primers, 0.5 umol/L PNA and certain amount of DNA. The PNA sequence was 5′-CCTACGCCACCAGCTCC-3′. The forward primer sequence was 5′-GCCTGCTGAAAATGACTGAATATAA-3′ and the reverse primer was 5′-biotin-CGTCAAGGCACTCTTGCCTAC-3′. Thermocycling was performed in an Mx3000P (Stratagene) real-time PCR instruction and the cycling conditions were as follows:98°C for 30 s and then 40 cycles of 98°C for 10 s, 72°C for 10 s, 62°C for 20 s and 72°C for 20 s. Pyrosequencing was performed in Pyrosequencing PSQ96 HS System (Biotage AB). The sequence of pyrosequencing primer was 5′-TGTGGTAGTTGGAGCT-3′. Nucleotide dispensation order was cyclic TACG from 5′ to 3′.

### Patient Population and Treatment Regimens

We retrospectively analyzed 35 patients with histologically confirmed mCRC with KRAS mutation from 2006 to 2010. All tumors were colorectal adenocarcinomas. Patients gave informed written consent and were treated with cetuximab or ceruximab-based regimens. Cetuximab was administered as a single agent or in combination with chemotherapy-based regimens with the same dose until disease progression. Cetuximab was administrated at a dose of 250 mg/m^2^ (initial dose was 400 mg/m^2^). There were 28 patients who received cetuximab alone. There were 4 patients who received 5-Fu treatment plus cetuximab treatment after failure of 5-Fu therapy. Another 3 patients received irinotecan, 5-Fu and cetuximab therapy after disease progress of irinotecan plus 5-Fu therapy.

### Clinical Evaluation and Tumor Response Criteria

The clinical response was assessed every 2 months by computer tomography (CT) scan of the chest and abdomen and/or magnetic response scan (MR). The Response Evaluation Criteria in Solid Tumor (RECIST) version 1.0 were adopted for clinical evaluation. According to RECIST version 1.0, complete response (CR) was defined as disappearance of all target lesions; partial response (PR) was at least a 30% decrease in the sum of the longest diameters (SLD) of target lesions taking as reference the baseline SLD; progressive disease (PD) was at least a 20% increase in the SLD of target lesions, taking as reference the smallest SLD recorded since the treatment started; stable disease (SD) was neither sufficient decrease in SLD to qualify for PR nor sufficient increase in SLD to qualify for PD. Two oncologists and radiologists verified the clinical response for all patients in a blinded manner.

### Statistical Analysis

Patients with CR, PR and SD were defined as patients with disease control and the diseased control rate was calculated as the number of disease control patient/the number of all patients. Chi-square test was carried out to compare the disease control rate of different groups in the 35 colorectal patients. The Chi-square was carried out by SPSS 16.0. A p value of <0.05 was considered statistically significant.

## Results

### Validation of PNA-PCR Method on Cell Lines

**Figure 1:**
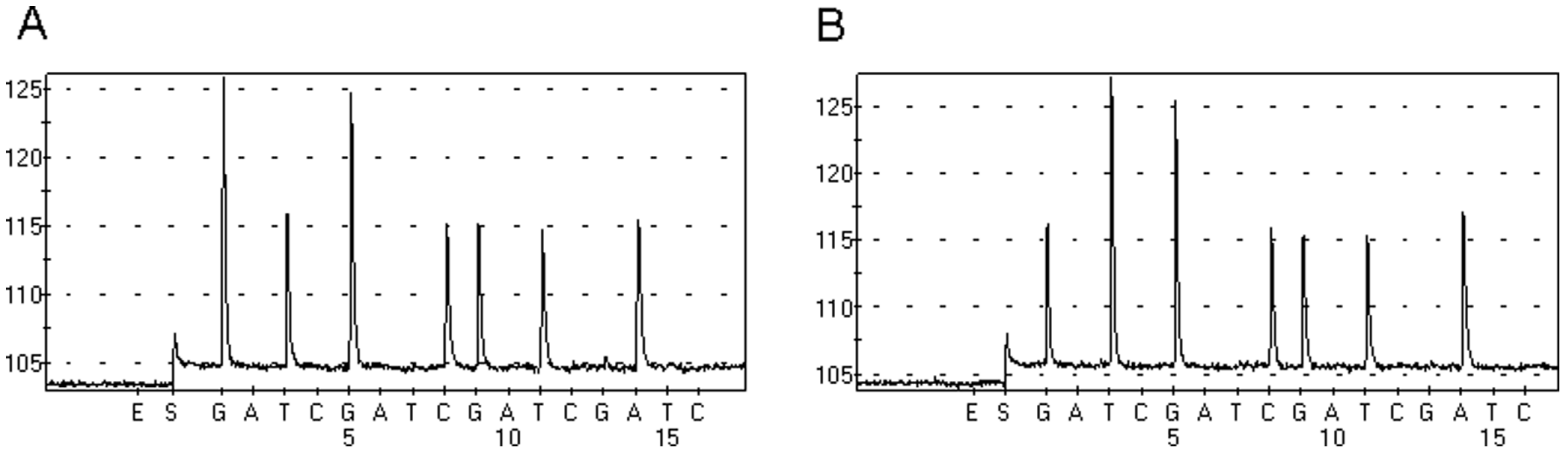
PNA-PCR assay could suppress wild-type DNA more completely in the existence of KRAS mutant DNA. (A) There are wild-type PCR products when adding 100 ng KRAS wild-type DNA in PNA-PCR assay. (B) There are only KRAS mutant PCR products when adding 100 ng wild-type DNA and 0.01 ng KRAS mutant DNA.

We found that when we added 100 ng KRAS wild-type DNA in the PNA-PCR assay, the PNA could suppress the amplification of wild-type DNA. However, this suppression was not complete and stable after repeated tests (Fig. 1A). However, when we added 100 ng KRAS wild-type DNA and 0.01 ng KRAS mutant DNA in PNA-PCR assay, the KRAS wild-type DNA could be completely and stably suppressed and only KRAS mutant DNA was amplified after confirmation of pyrosequencing (Fig. 1B), which indicated that even the mutant DNA was rare comparing to wild-type, PNA assay still amplify mutant DNA exclusively and this amplification could assist to suppress the wild-type DNA amplification completely.

In order to make sure the stability of the assay, we decreased the amount of wild-type DNA to 10 ng added in the assay and found that PNA could completely and stably suppress the amplification of wild-type DNA even in the absence of KRAS mutant DNA (no PCR products, confirmation by gel electrophoresis and pyrosequencing, data not shown). Therefore, in the following experiment, we controlled the DNA added in the assay no more than 10 ng.

### Validation of PNA-PCR Method on Cancer Patients’ Tumor Tissues

**Figure 2:**
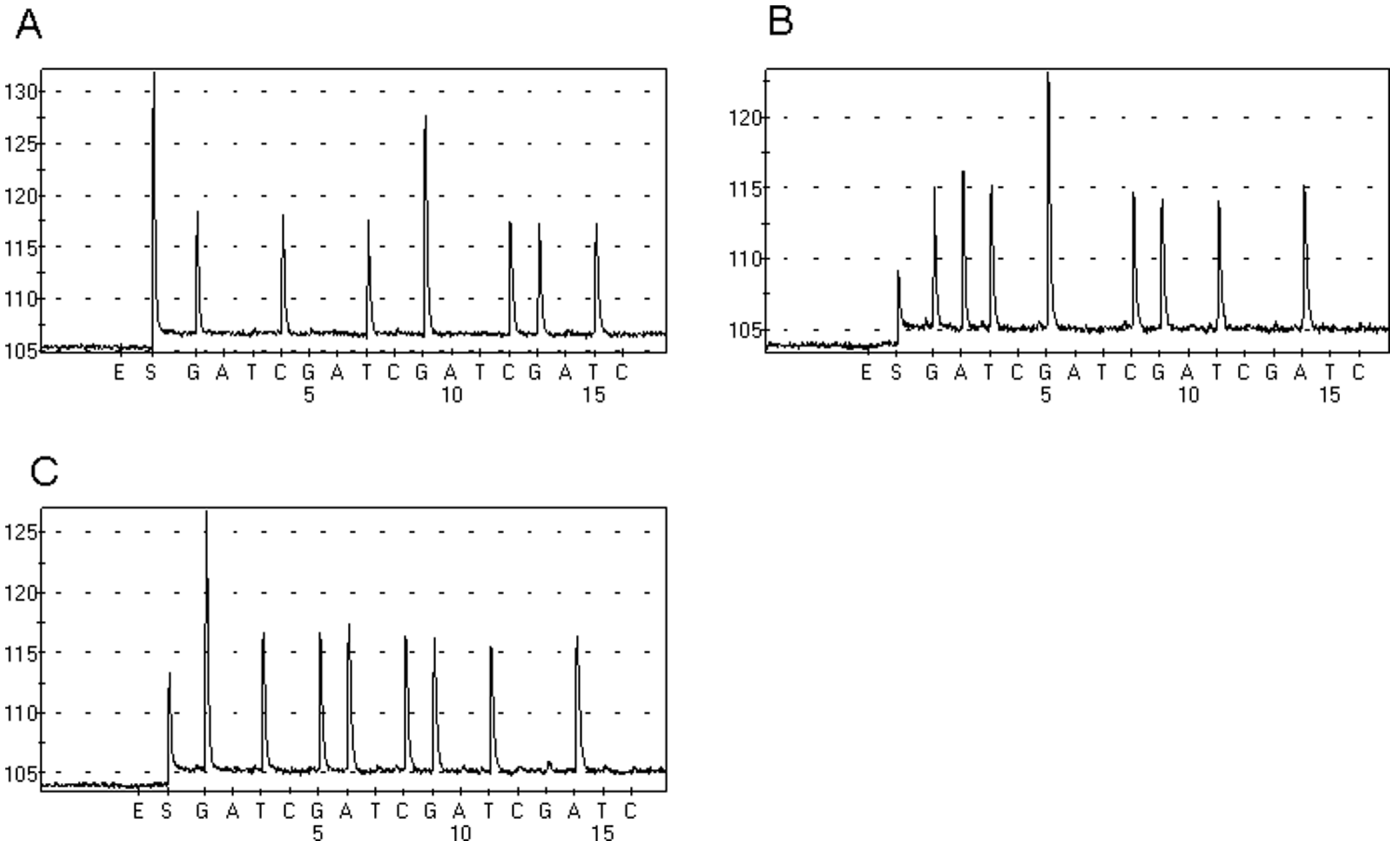
PNA-PCR amplified KRAS mutant DNA of FFPE sample exclusively. A Tumor tissue contains a GGT→GCT mutation. B Tumor tissue contains a GGT→GAT mutation. C Tumor tissue contains a GGC→GAC mutation.

A total of 47 colorectal cancer patients were confirmed to carry KRAS mutation by COLD-PCR/Sanger sequencing [17]. In total, there were 15 G12D, 13 G12V, 13 G13D, 2 G12S, 2G12A, 2G13R and 1G12C KRAS mutations (one patient carrying two types of KRAS mutations). The percentage of tumor cell in FFPE samples was listed in table 1. No more than 10ng DNA of FFPE sample was added to PNA-PCR assay and the PCR products were sequenced by Pyrosequencing. As we expected, the PNA-PCR assay amplified KRAS mutant DNA exclusively. Figure 2 shows that no wild-type DNA was amplified in PNA-PCR assay.

**Table 1:** Calculation of KRAS mutation abundance in tumor cells.

Subject number	KRAS mutation (Sanger sequencing)	KRAS mutation (PNA-PCR/prosequencing)	Tumor cell percentage in tumor tissue (%)	Mutant DNA percentage in total DNA (%)	Mutant DNA percentage in tumor cells (%)
1	GGT→GTT	GGT→GTT	60	33.4	55.7
2	GGC→GAC	GGC→GAC	20	18	90.0
3	GGT→GAT	GGT→GAT	30	20.4	68.0
4	GGT→GTT	GGT→GTT	40	21.6	53.5
5	GGT→GAT	GGT→GAT	50	10.0	20.0
6	GGT→GAT	GGT→GAT	75	36.6	48.8
7	GGT→GTT	GGT→GTT	55	19.6	35.6
8	GGT→GAT	GGT→GAT	40	21.3	53.3
9	GGT→GAT	GGT→GAT	40	11.3	28.3
10	GGT→AGT	GGT→AGT	70	28.5	40.7
11	GGT→GTT	GGT→GTT	70	12.2	17.4
12	GGT→GAT	GGT→GAT	70	19.3	27.6
13	GGT→GAT	GGT→GAT	68	11.7	17.2
14	GGT→GAT	GGT→GAT	70	39.8	56.9
15	GGC→GAC	GGC→GAC	65	20	30.8
16	GGT→GTT	GGT→GTT	65	14.4	22.2
17	GGC→GAC	GGC→GAC	60	57.8	96.3
18	GGT→GTT	GGT→GTT	65	49	75.4
19	GGT→TGT	GGT→TGT	50	5.4	10.8
20	GGC→GAC	GGC→GAC	40	8.7	25.5
21	GGT→GAT	GGT→GAT	25	8.4	33.6
22	GGT→GAT	GGT→GAT	30	4.7	15.7
23	GGT→GTT	GGT→GTT	60	20.6	34.3
24	GGT→GCT	GGT→GCT	40	28.9	72.3
25	GGT→GAT	GGT→GAT	35	16.6	47.4
26	GGC→GAC	GGC→GAC	25	20.9	83.6
27	GGT→GCT	GGT→GCT	60	49.0	81.7
28	GGT→GTT	GGT→GTT	40	18.9	47.3
29	GGC→GAC	GGC→GAC	35	34.4	98.3
30	GGC→GAC	GGC→GAC	30	15.2	50.7
31	GGT→AGT	GGT→AGT	50	30.1	60.2
32	GGT→GTT	GGT→GTT	50	21.6	43.2
33	GGC→GAC	GGC→GAC	50	44.4	88.8
34	GGT→GAT	GGT→GAT	55	23.3	42.4
35	GGT→GTT	GGT→GTT	70	23.7	33.9
36	GGC→GAC	GGC→GAC	45	26.4	58.7
37	GGT→GTT	GGT→GTT	45	37.4	83.1
38	GGC→GAC	GGC→GAC	60	19.3	32.2
39	GGC→TGC	GGC→TGC	50	40.9	81.8
40	GGT→GTT	GGT→GTT	70	17.1	24.4
41	GGT→GTT	GGT→GTT	60	46.3	77.2
42	GGC→TGC GGC→GAC	GGC→TGC GGC→GAC	55	28.9	52.5
43	GGC→GAC	GGC→GAC	60	37.1	61.8
44	GGC→GAC	GGC→GAC	30	22.4	74.7
45	GGT→GAT	GGT→GAT	40	38.7	96.8
46	GGT→GAT	GGT→GAT	55	50	90.9
47	GGT→GAT	GGT→GAT	60	26.6	44.3

### Detect and Calculate the Percentage of Mutant KRAS DNA

**Figure 3:**
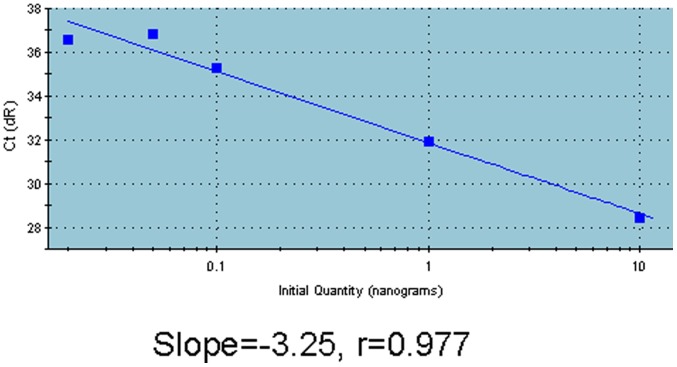
Quantification of KRAS mutant DNA. Varying amounts of KRAS mutant DNA were plotted against Ct values (threshold cycle). Slope, r value and regression line are shown.

Before detecting the percentage of mutant DNA of tumor tissue, we analyzed the standard curve of the PNA-PCR assay and found the regression line was linear (slope = −3.25; r = 0.977) in the range of 0.02 to 10 ng of KRAS mutant DNA (Fig. 3), which means this assay could quantify KRAS mutant DNA.

No more than 10 ng DNA from FFPE tissue (2 ul) was added to PNA-PCR assay and regular PCR assay (without PNA) respectively (all reactions were run in triplicate). One sample’s total DNA and KRAS mutant DNA could be read by standard curve. The DNA amount in regular PCR assay was total DNA as all kinds of DNA could be equally amplified and the DNA amount in PNA-PCR assay was KRAS mutant DNA as the wild-type one didn’t amplify in this assay. The percentage of KRAS mutant DNA was calculated as amount of KRAS mutant DNA/amount of total DNA. The percentage of KRAS mutant DNA in tumor cells was calculated as percentage of KRAS mutant DNA in total DNA/percentage of tumor cells in tumor tissue. The percentage of mutant DNA in tumor cells varied from 10.8% to 98.3% (Table 1). In total, there were 10 samples containing mutant DNA less than 30%, 27 samples from 30% to 80% and 10 samples more than 80%.

### Percentage of KRAS Mutation in Tumor Cells and Correlation to Response

Patient’s characteristics and percentage of KRAS mutation in tumor cells have been listed in table 2. The rate of partial response was 2.9% (1/35), stable disease 14.3% (5/35) and progressive disease 82.9% (29/35). Correlation between percentage of KRAS mutation in tumor cells and disease control was listed in table 3. In total, in the KRAS mutation less than 30% group the disease control rate was 44.4%; the disease control rate of 30∼80% group was 5.6% and the >80% group was 12.5% (P = 0.038). It was worthy to note that in the >80% group the patient who had stable disease contains a G13D mutation. In this study, the response rate was 18.5% (5/27) for KRAS 12 codon mutations and 12.5%(1/8) for KRAS G13D mutation (Table 4). We didn’t observe that patients with KRAS G13D mutation had better response rate (Table 4).

**Table 2:** Patients’ characteristics of the 35 patients receiving cetuximab.

Parameter	Number (n = 35)	Percentage
Age		
Median (range)	65 (40–76)	
Gender		
Female	15	42.9%
Male	20	57.1%
PS at inclusion		
0	8	22.9%
1	15	42.9%
2	12	34.3%
Locus primary tumor		
Rectum	18	51.4%
Colon	17	48.6%
Previous surgery for primary		
Yes	30	85.7%
No	5	14.3%
No of metastatic sites		
1–2	20	57.1%
>2	15	42.9%
Treatment		
Cetuximab alone	28	80.0%
Cetuximab+5-Fu	4	11.4%
Cetuximab+5-Fu +irinotecan	3	8.6%
Type of KRAS mutation		
G12V	12	34.3%
G12D	10	28.6%
G13D	8	22.9%
G12A	3	8.6%
G12C	2	5.7%
G12S	1	2.9%
Percentage mutant KRAS in tumor cells		
<30%	9	25.7%
30%∼80%	18	51.4%
>80%	8	22.9%

**Table 3:** Correlation between percentage of KRAS mutation in tumor cells and disease control rate.

Best response	Total	<30%	30∼80%	>80%
Disease control (CR+PR+SD)	6 (17.1%)	4 (44.4%)	1 (5.6%)	1 (12.5%)
PD	29 (82.9%)	5 (55.6%)	17 (94.4%)	7 (87.5%)
Total	35	9	18	8

**Table 4:** Patients’ mutation type and response to therapy.

Mutation type	G12D	G12V	G12A	G12C G12C/G12S	G13D
Patients’ total number	10	12	3	2	8
Patients’ number with disease control	2	3	0	0	1
Response rate of different mutation type	20.0%	25%	0%	0%	14.3%

## Discussion

The present study proved that PNA-PCR method could quantify and calculate the KRAS mutation’s abundance of tumor cells. As far as we know, we firstly showed that low abundance of KRAS mutation (<30%) might also benefit from anti-EGFR treatment in metastatic colorectal cancer patients. Since previous studies only focused on whether the mutation was positive, our study revealed a new research concept of EGFR antibody therapy.

In this study, there were 7 patients treated by both cetuximab and chemotherapy. However, all these 7 patients received cetuximab after failure of original chemotherapy. Therefore, we tended to believe that patients’ response to therapy mainly contributed to cetuximab. Although there were studies proved that patients with KRAS G13D mutation showed a strong trend to a more favorable outcome comparing to codon 12 mutations, we failed to observe this in our study potentially because of the limited number patients of this study.

Our results indicated that the percentage of KRAS mutant DNA in tumor cells varied from 10.8% to 98.3% and this distribution was continuous. As we expected, patients bearing low abundance (<30%) of KRAS mutation had higher disease control rate to cetuximab than other groups (44.4% for <30% group vs 5.6% for 30∼80% group and 12.5% for >80% group, P = 0.038). If we excluded a disease control patient who carried a G13D mutation in >80% group, the trend that low abundance had higher disease control rate was more obvious.

Although direct Sanger sequencing was regarded as a classic method for screening KRAS mutations, more and more high sensitivity methods such as COLD-PCR, ARMS, mutant-enriched PCR, competitive amplification of differentially melting amplicons (CADMA) [18], [19], [20], [21] were applied to screen KRAS mutations. These methods assisted us to find more KRAS mutant samples. However, whether these samples could benefit from anti-EGFR therapy remains unknown. Our study showed that these samples with low abundance of KRAS mutations might benefit from EGFR antibody therapy.

Why did tumor with low abundance of KRAS mutation tend to response to EGFR antibody therapy? One potential explanation was that tumor cells had great heterogeneity. When the KRAS mutant tumor cells were minority, most of KRAS wild-type tumor cells could also be killed by EGFR antibody treatment and in this process cancer patients could show a relatively long time of stable disease or even partial response. However, as therapy continues, more and more KRAS wild-type tumor are wiped out, the abundance of KRAS mutation becomes higher and patients tend to have a progression disease and become resistant to EGFR antibody therapy.

These could also explain why EGFR antibody treatment is initially effective to most KRAS wild-type cancer patients and then losts its efficiency as therapy continues. A recent article published in *Nature* found that 60% of patients who developed resistance to EGFR antibody treatment showed acquisition of secondary KRAS mutations [22]. We tend to believe that this “secondary” KRAS mutation actually exists in primary tumor tissues. Since tumor cells containing this mutation were too few, we couldn’t detect them before therapy. However, as therapy continued, the proportion of tumor cells adjusted under the therapy pressure. In this situation anti-EGFR therapy lost its efficiency and the “secondary” KRAS mutations could also be easily detected.

More importantly, Misale et al also found that KRAS mutant alleles could be detected in the blood of anti-EGFR treatment patients as early as 10 months before radiographic documentation of disease progression [22]. In other words, even the KRAS mutant alleles exist, patients could still have a relative long time (10 months) of stable disease. This article’s result coincides with our findings and shows that existence of KRAS mutations doesn’t mean a complete resistance.

Although some researchers had paid attentions to distinguish low and high abundance of EGFR mutation recently [15], our advantage was more obvious as our method could easily and accurately calculate the precise percentage of KRAS mutant alleles. This precise calculation could reflect the percentage of KRAS mutation more objectively and assist other laboratory verify our conclusion more easily.

In summary, our study demonstrated that PNA-PCR method could easily detect the percentage of KRAS mutation in tumor cells. Colorectal cancer patients with low abundance of KRAS mutation might benefit from EGFR antibody therapy and further research is needed on a larger number patients.
